# Comparing three approaches to delirium identification in older patients presenting to the emergency department

**DOI:** 10.1007/s41999-026-01612-w

**Published:** 2026-10-05

**Authors:** Maaike A. Pouw, Eline G. Haan, Carolien J. Jansen, Monika Trzpis, Barbara C. van Munster

**Affiliations:** 1 Department of Geriatric Medicine, University of Groningen, University Medical Center Groningen, Hanzeplein 1, 9700 RB Groningen, The Netherlands; 2 Department of Geriatric Medicine, Nij Smellinghe Drachten, Compagnonsplein 1, 9202 NN Drachten, The Netherlands; 3 Department of Geriatric Medicine, Martini Hospital Groningen, Van Swietenlaan 1, 9728 NT Groningen, The Netherlands

**Keywords:** Delirium, Screening, Emergency department

## Abstract

**Introduction:**

Early identification of hospitalised patients with or at risk for delirium is important to treat patients adequately. The aim of this study is to compare three approaches to delirium identification for identifying patients being at risk for or with a delirium during hospitalisation in a cohort of older patients presenting to the emergency department, including retrospectively extracted indicators of delirium based on prehospital care notes, by risk assessment (VMS), and screening instrument (4AT).

**Methods:**

A single-centre cohort study was conducted with the use of data from patients 70 years and older from Acutelines, a hospital-based biobank. extended with extraction of documented prehospital indicators based on prehospital care notes and a hospital chart-based review as a reference standard to determine whether patients experienced delirium during hospitalisation. We compared the different approaches by calculating standard performance measures (e.g. sensitivity, specificity, and predictive values).

**Results:**

Data of 655 patients were included. Median age was 76 years, and 407 (62%) were male. During hospitalisation, 146 patients (22%) were identified as having a delirium. The positive likelihood ratios were 5.54 for the documented prehospital indicators, 1.55 for the VMS risk assessment, and 2.39 for the 4AT screening.

**Conclusion:**

All three approaches, the documented prehospital indicators, the VMS risk assessment, and 4AT screening, are associated with delirium during hospitalisation. The documented prehospital indicators could be of added value, and we see this as a starting point for research into how the prehospital information could be used in the acute care pathway for older patients with or at risk for delirium.

**Supplementary Information:**

The online version contains supplementary material available at https://doi.org/10.1007/s41999-026-01612-w.

## Key summary points

**Aim:**

To compare three approaches of delirium identification—retrospectively extracted documented prehospital indicators of delirium, a risk assessment (VMS), and a screening (4AT)—in identifying older patients presenting to the emergency department at risk for or experiencing delirium during hospitalisation.

**Findings:**

Among 655 patients aged 70 years and older, 22% developed delirium during hospitalisation. All three approaches were associated with delirium presence, with the documented prehospital indicators showing a positive likelihood ratio of 5.54, the VMS risk assessment 1.55, and the 4AT screening 2.39.

**Message:**

The documented prehospital indicators could be of added value, and we see this as a starting point for research into how prehospital information could be used in the acute care pathway for older patients with or at risk for delirium.

**Supplementary Information:**

The online version contains supplementary material available at https://doi.org/10.1007/s41999-026-01612-w.

## Introduction

Delirium is an acute disturbance in attention, awareness, and cognition, as a direct consequence of a physiological event such as an acute disease [1]. The prevalence and incidence of delirium in acute medical adult inpatients is about one in four older patients, and these numbers have remained broadly stable in the past decades [2, 3]. The presence of delirium should prompt healthcare professionals to search for the underlying illness or cause and treat accordingly. Besides causing distress in patients and carers, delirium is often a sign of vulnerability and is associated with adverse outcomes of hospitalisation, including increased mortality, longer length of hospital admission, long-term cognitive decline, poorer functional status, and institutionalisation [2, 4–6]. An active approach in identification of delirium pays off, as higher screen rates are associated with fivefold higher recognition rates, demonstrating the need for screening in clinical practice [5]. Identifying older patients at risk of a delirium is of importance for prompt introduction of intervention strategies. In hospitalised patients up to a third of incident delirium could be prevented through multicomponent interventions [7–9].

Although the awareness of delirium is rising, the diagnosis of delirium is frequently overlooked, especially in settings such as ambulances or in an emergency department (ED) [10, 11]. Various ways of delirium assessments have been developed, validated, and implemented in the past decades [8, 12]. These assessments can be divided into risk factor-based assessments, which focus on estimating the risk of developing delirium, and feature-based assessments, which focus on identifying the current presence of delirium, with some degree of overlap between the two. In Dutch hospitals, a delirium risk screening has been introduced in 2012 for patients over 70 years with the VMS delirium score, which identifies patients at risk of developing delirium [13, 14]. A widely used feature-based instrument is the 4AT, which screens for the presence of delirium at the time of assessment [15]. Most delirium assessments are designed for hospital setting, with scarce literature on use or validation in prehospital care [16]. For example, systematic reviews on the diagnostic test accuracy of the 4AT did not include any studies conducted in a prehospital setting [17, 18]. Primary care professionals often rely on clinical impression, using patient observations, history, and caregiver input. Sands et al. suggest that asking, *"Has [patient’s name] been more confused lately?"* can detect 80% of delirium cases, highlighting the value of clinical impression [19].

The aim of this study is to compare three approaches to delirium detection for identifying patients being at risk for or with the presence of a delirium during hospitalisation in a cohort of older patients presenting to the emergency department, including; retrospectively extracted indicators of delirium based on prehospital care notes, a risk factor-based assessment (VMS delirium score), and a feature-based screening instrument (4AT).

We aimed to improve understanding of delirium detection in routine clinical practice and to inform efficient use of healthcare resources.

## Methods

### Setting, study population, and study design

This single-centre, cohort study was conducted with use of data from the Acutelines databank from September 2020 to February 2024. Acutelines is a data, image, and biobank that includes patients with a spectrum of acute conditions, triaged with higher urgency codes (emergency, very urgent, or urgent) and/or specific conditions such as sepsis, shock, or venous thrombosis, initiated by the departments of Emergency Medicine and Internal Medicine of the University Medical Center Groningen (UMCG), the Netherlands [20]. For the inclusion in the Acutelines databank, all patients visiting the ED of the UMCG were screened for eligibility by the ED (triage)nurse together with a trained research assistant and consent (by proxy) was obtained. Data were collected from hospital electronic health records or directly from bedside monitors, and assessments were used to obtain information regarding health status, frailty, and cognitive function. From the Acutelines database, we received a dataset of all included patients aged 70 years and older at the time of inclusion. From this dataset, all patients admitted for at least one night after the ED visit and with a completed 4AT assessment at admission were included in this study. In case of subsequent ED visits for the same patient, the first visit of each patient was included. Availability of the 4AT was selected as an inclusion criterion because of its availability in the dataset and its ability to be completed in all patients, including those who were unable to respond.

### Study variables and outcomes

Data were collected on demographic characteristics (age, gender, marital status, living situation, and educational level), frailty (assessed through the Acutely Presenting Older Patient (APOP) score), and comorbidity level, evaluated using the Charlson Comorbidity Index (CCI)) [21, 22].

Data were collected, processed, and archived in accordance with the General Data Protection regulation (GDPR) and the FAIR (Findable, Accessible, Interoperable, Reusable). The Acutelines study data were collected and managed using REDCap electronic data capture tools hosted at University Medical Center Groningen. REDCap (Research Electronic Data Capture) is a secure, web-based software platform designed to support data capture for research studies. The system is compliant with good clinical practice and ISO 27001 certified [23, 24].

The primary outcome of this study is delirium occurrence during hospitalisation and is considered positive when delirium or delirium features based on DSM-5 criteria (Supplementary Appendix 1) are mentioned in the hospital chart. Data regarding the referral documentation and hospital records were reviewed on a case-by-case basis retrospectively. An additional check was performed to verify if delirium was not missed by checking if there was new antipsychotic prescription or at least one Delirium Observation Screening Scale (DOSS) score of 3 or higher during hospitalisation, similar to previous described chart reviews for delirium [25], Two different assessors (EH and CJ) did the reviewing of the hospital electronic health records; each file was assessed by one assessor. Before the start, five cases were independently assessed by three different assessors (EH, CJ, MP), then discussed and compared in order to reach consensus. In case of uncertain evaluation by a first assessor, the respective file was discussed in a consensus meeting with the third assessor (MP) and consensus was established through discussion of information and interpretation.

The secondary outcomes are retrospectively extracted documented prehospital indicators of delirium, VMS delirium risk score, 4AT-score, and delirium occurrence during hospitalisation [15, 26]. The documented prehospital indicators of delirium were retrospectively determined by reviewing the free-text documentation (referral notes, handovers) of general practitioners and ambulance professionals. Documentation was considered positive when the notes contained an explicit suspicion of delirium or descriptions considered consistent with delirium (including progressively confused, disoriented, incoherent). The VMS delirium risk score is part of the Dutch national VMS guidelines (VMS, veiligheidsmanagementsysteem translates to safety management system guidelines) and is a three-question tool (*memory problems, in need for self-care, previous delirium*) to identify patients at risk for development of delirium [13, 14]. The VMS is assessed at the emergency department by a nurse or research assistant. The 4AT is a widely used delirium assessment tool to detect delirium at the time of assessment. It requires no special training and can assess drowsy or agitated patients. It includes four items: alertness, the Abbreviated Mental Test-4 (AMT4), attention testing (months backward), and mental status changes [15]. The 4AT is administered at the emergency department by a nurse or research assistant.

According to the predefined cutoffs, a VMS delirium score ≥1 or a 4AT score ≥4 was considered positive (Supplementary Appendices 1 and 2). The documented prehospital indicators of delirium were considered positive when the free-text documentation (referral notes, handovers) of general practitioners and ambulance professionals contained an explicit suspicion of delirium or descriptions considered consistent with delirium.

### Data analysis

Statistical analysis was performed using IBM SPSS Statistics, version 28.0.1.0 (IBM Corp, Armonk, New York, USA). Continuous data were expressed as the mean ± standard deviation (SD), with 95% confidence intervals for normally distributed variables, or median + interquartile range (IQR) for non-normally distributed variables. Categorical data were expressed as frequencies and proportions.

All approaches to delirium identification and delirium during hospitalisation had a dichotomous outcome, positive or negative for delirium, based on cutoff values (≥ 1, and ≥4 for VMS and 4AT, respectively) or positive note of referral/ambulance or positive chart review [15, 26]. The sensitivity, specificity, positive predictive value (PPV), negative predictive value (NPV), positive likelihood ratio, and negative likelihood ratio with 95% confidence intervals were calculated, and receiver operating curves were constructed. The compliance to VMS screening was calculated using descriptive statistics and expressed as frequency and proportion.

### Reporting

This study complies with the Standards for Reporting of Diagnostic Accuracy Studies guidelines, the STARD, 2015 [27]. (Supplement 4) Although the primary objective of the present study was not to evaluate diagnostic test accuracy, we considered the STARD guideline to be the most appropriate reporting framework for this study.

## Results

**Fig. 1:**
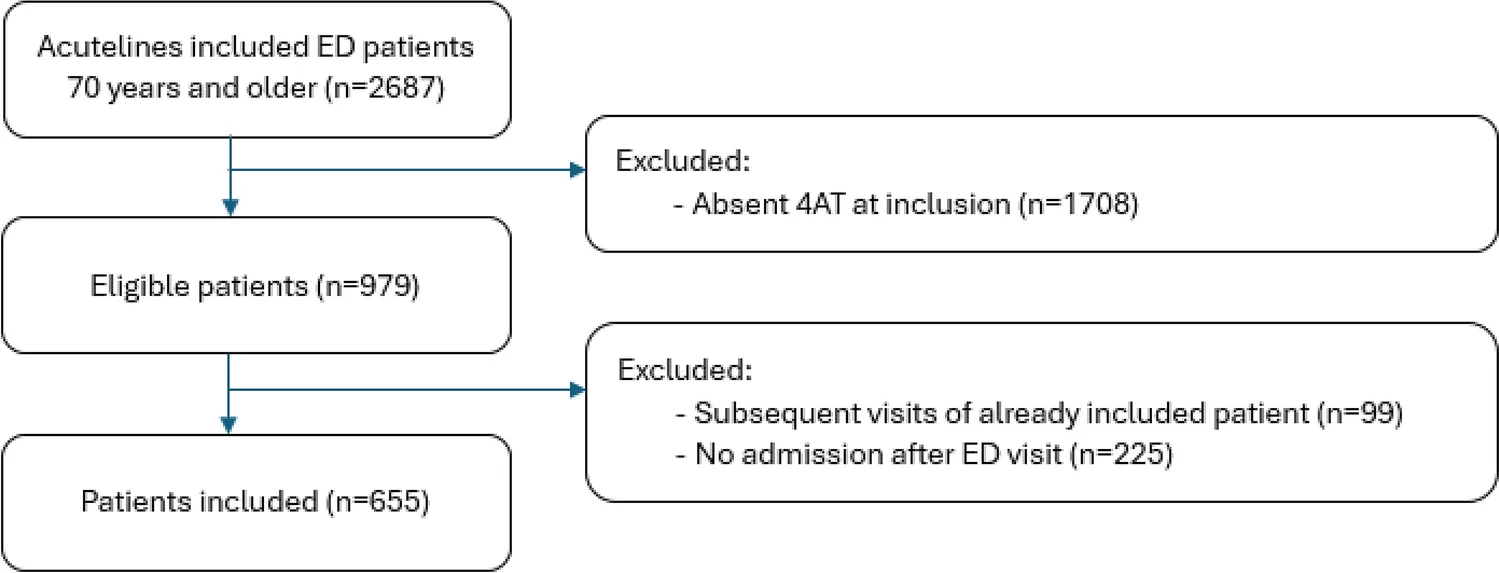
Flowchart of patient selection in cohort

A total of 655 patients were included in the cohort (Fig. 1, flowchart). Patient characteristics are shown in Table 1. The characteristics of patients with or without a completed 4AT are shown in Supplementary Table 5; missingness of 4AT leading to exclusion reflects operational factors more than patient-related factors. Median age was 76 (IQR 73–81), and 407 patients (62%) were male. Delirium during hospitalisation occurred in 146/655 patients (22%).

**Table 1:** Patient characteristics

	All participants (*n* = 655)	Delirium during admission (*n* = 146)	No delirium during admission (*n* = 509)
Age, median (IQR)	76 (74–82)	76 (74–82)	76 (74–82)
Male gender, no. (%)	407 (62)	108 (74)	299 (59)
Marital status (*n* = 638)			
Unmarried/widowed, *n* (%)	229 (35)	51 (35)	178 (35)
Married, *n* (%)	409 (62)	89 (64)	320 (64)
Living situation (*n* =646)			
Living alone, *n* (%)	214 (33)	50 (35)	164 (33)
Living together, *n* (%)	396 (61)	82 (57)	314 (62)
Care facility *n* (%)	36 (6)	11 (8)	25 (5)
Level of education (*n* = 611)			
Intermediate/lower education, *n*(%)	464 (76)	96 (73)	368 (77)
Higher education, *n* (%)	147 (24)	35 (27)	112 (23)
Charlson comorbidity index (CCI)	5 (4–7)	5 (4–7)	5 (4–7)
APOP score, median (IQR)	26,5 (18–37)	29 (21–49)	26 (17–36)
APOP: diagnosed with dementia, *n* (%)	16 (2.4)	7 (5)	9 (2)

*IQR* interquartile range, *APOP* *score* (acutely presenting older patient) as indication for frailty score and risk of functional decline percentage, and delirium based on DSM-5 criteria

A total of 531 patients (81%) had a documented prehospital clinical impression in a referral or ambulance note. The number of delirium cases is 128/531 (24.1%). The number of positive documented prehospital indicators of delirium is 69/531 (13%), of which 44 were confirmed delirium during hospitalisation.

A VMS screening was available for 614 patients (94%). The number of delirium cases was 134/614 (21.5%). The number of positive VMS scores was 335/531 (63%), of which 104 were confirmed delirium during hospitalisation. A 4AT screening was available for 655 patients (100%). The number of delirium cases was 146/655 (22.3%). The number of positive 4AT assessments was 145/655 (22%), of which 59 were confirmed delirium during hospitalisation. The positive likelihood ratios of the documented prehospital indicators of delirium, VMS, and 4AT were 5.54, 1.55, and 2.39. The negative likelihood ratios were 0.70 for the documented prehospital indicators of delirium, 0.48 for VMS screening, and 0.72 for 4AT. The positive predictive values were 0.64 for the clinical impression, 0.31 for the VMS, and 0.83 for the 4AT. Table 2 presents the performance metrics for each approach. ROC curves are provided in Supplement 6, and the overlap in positive classifications across the different delirium identification approaches is presented in Supplementary Table 7.

**Table 2:** Performance metrics of the different approaches: documented prehospital indicators of delirium, VMS, and 4AT

	Documented prehospital indicators of delirium *N* = 531	95% CI	VMS *N* = 614	95% CI	4AT *N* = 655	95% CI
TP (*n*)	44		104		59	
FP (*n*)	25		231		86	
TN (*n*)	378		245		423	
FN (*n*)	84		34		87	
Total (*n*)	531		614		655	
Sensitivity	0.34	0.27–0.43	0.75	0.68–0.82	0.41	0.33–0.49
Specificity	0.94	0.91–0.96	0.52	0.47–0.56	0.83	0.80–0.86
PPV	0.64	0.52–0.75	0.31	0.26–0.36	0.83	0.80–0.86
NPV	0.82	0.79–0.85	0.88	0.84–0.91	0.40	0.33–0.48
PLR	5.54	3.54–8.68	1.55	1.36–1.77	2.39	1.81–3.15
NLR	0.70	0.62–0.80	0.48	0.35–0.65	0.72	0.62–0.82
AUC	0.64	0.58–0.70	0.63	0.58–0.69	0.62	0.56–0.67
OR	7.92	4.59–13.66	3.2	2.12–4.97	3.36	2.23–5.00

*95%CI* 95% confidence interval, *TP* true positives, *FP* false positives, *TN* true negatives, *FN* false negatives, *PPV* positive predictive value, *NPV* negative predictive value, *PLR* positive likelihood ratio, *NLR* negative likelihood ratio, *AUC* area under the curve, *OR* odds ratio

Information on APOP-frailty scores, comorbidity, and if patients had a known diagnosis of dementia based on APOP-screening question is provided in Table 1. The APOP known diagnosis of dementia was positive in only 16 cases, 7 in patients with delirium (5%) and 9 in patients without delirium (2%).

In case of uncertain evaluation by a first assessor on the documented prehospital indicators of delirium, or uncertainty if the hospital chart was positive for delirium, a consensus meeting agreement was established through discussion of information and interpretation. Discussion took place in 134 of 655 cases (20%). Examples that prompted discussion included: cases of dementia rather than delirium, which was initially not known to the care providers; or regarding preventively prescribed delirium medication that had not been administered, given that no delirium had occurred. Examples of information from hospital charts are shown in Supplementary 8. When reviewing the VMS scores, two types of discrepancies were identified: (1) the screening had been completed twice at different time points, yielding inconsistent responses; and (2) the screening indicated that the patient had never experienced delirium, whereas a previous episode of delirium was documented in the patient’s medical history. Discrepancies were present in 78 of 655 cases (12%).

## Discussion

The prevalence of delirium in this cohort (22%) was similar to previous studies with prevalences of approximately one in four older patients [3, 8, 28]. Male gender and frailty (APOP score percentage) seem overrepresented in the delirium cohort compared to the non-delirium cohort, in line with existing literature on gender disparities and frailty in delirium risk [3, 29–31].

We compared three approaches to delirium identification early in the acute care pathway and their association with delirium during hospitalisation among older patients presenting to the emergency department. The positive likelihood ratio was 5.54 for the documented prehospital indicators of delirium, 2.39 for the 4AT, and 1.55 for the VMS. The low sensitivity (0.34) of the documented prehospital indicators of delirium may partly reflect documentation gaps rather than true absence of delirium at the time of prehospital contact. Among patients with negative documented prehospital indicators of delirium, 16% (84 false negatives) developed delirium during hospitalisation, similar to the 13% (87 false negatives) with a negative 4AT score. This likely reflects the timing of assessment, as delirium was monitored throughout the hospital stay and may not have been present initially; this is also the most plausible explanation for the poorer performance metrics, e.g. the low negative predictive value of 0.4, than expected from the 4AT [17]. In contrast, the VMS delirium score, based on risk factors rather than clinical features, produced more false positives (231 patients, 38%) but fewer false negatives (34 patients, 6%), suggesting a superior negative predictive value and a low positive predictive value (0.31) compared to the other approaches. A positive risk assessment does not necessarily mean that delirium will occur, resulting in a higher number of false positives. A higher false-positive rate may be acceptable however, as it increases the number of individuals receiving preventive care, regardless of whether the indication is ultimately justified, and may thereby contribute to prevention of delirium. In the context of screening, maximising sensitivity is of primary importance. The documented prehospital indicators of delirium demonstrate low sensitivity and are therefore less suitable as a screening tool; however, when findings are positive, they provide strong evidence for the presence of delirium.

Preferably, a risk factor-based score (VMS) should be validated against an optimal prediction model, while a feature-based score should be evaluated against the presence of DSM criteria. We recognise that the current study design is limited in this respect, as it entails comparing non-equivalent measures. However, the data used in the present study reflect routine clinical practice rather than a predefined trial setting. At present, there is no single optimal prediction model for delirium against which to compare [32]. This study is intended as an initial step toward better incorporating insights from the prehospital setting in the identification of delirium.

Although both the documented prehospital indicators of delirium and 4AT are feature-based approaches conducted around the same time, their overlap was limited. This discrepancy likely reflects the fluctuating course of delirium, differences in assessment timing, or early symptom resolution following interventions such as intravenous fluids or oxygen. The higher positivity rate of the 4AT may be related to its administration partly done by researchers, as research settings often yield higher detection rates—particularly when assessing alertness in acutely ill ED patients [28, 33]. In contrast, primary care or ambulance healthcare may underreport delirium features, especially in emergency contexts where physical stabilisation is prioritised [34]. As a result, delirium signs may be missed, not written down, overlooked, or not conveyed during hospital transfer.

We found a high VMS screening compliance rate of 94%, exceeding expectations on compliance of the assessment based on prior literature [13]. This is likely due to VMS screening becoming more established over time after an initial obligatory period, assessments being conducted by both ED staff and research assistants, and in some cases the assessment may have occurred after transfer from the ED, but always within the first 24 h. In the chart review, it was observed that for some patients, the VMS screening had been completed more than once. We consistently used the first completed screening; however, discrepancies were observed in the responses between different versions for the same patient or with the patient’s medical history, raising concerns about the test (retest) reliability. One possible explanation for this discrepancy is that the screening relies on patient self-report. Patients may not always recall a previous episode of delirium, particularly when the delirium occurred superimposed on dementia.

### Strengths and limitations

Strengths of this study include the sufficient sample size, a study in a realistic representation of routine ED practice, and multi-year data collection. Delirium occurrence was carefully assessed, with multiple reviewers evaluating records in uncertain cases (134/655 cases (20%)). For example, patients with Delirium Observation Screening Scale (DOSS) scores ≥3 but no other delirium features were discussed in consensus meetings with the third assessor, as scores could sometimes be attributed to alternative causes (e.g. cerebrovascular events) and not classified as delirium. A limitation is that the 655 medical records were not independently assessed by two reviewers, precluding calculation of Cohen’s kappa.

Another study limitation is due to the retrospective nature of the study; outcome parameters were limited to the ones that could be collected from the electronic patient records. Similar to other studies, we used health records as a basis for the outcome of delirium presence during admission [25, 33]. However, adequate health record notification depends on the recognition by the initial care provider, especially regarding those departments where staff are less experienced with delirium, which could lead to underreporting resulting in missed diagnosis of delirium. Additionally, reliance on written documentation has a limitation, as information communicated verbally in the ED may not be captured if it is not formally recorded. For instance, observations such as a patient appearing confused or exhibiting motor agitation may be communicated verbally, but omitted from the final medical record. Delirium remains a transient and fluctuating syndrome with heterogeneity of presentation across subtypes and can be difficult to recognise [4]. Furthermore, a notable number of clinical impressions from prehospital care were missing due to patient self-referral or direct admission through their hospital specialists. Despite this, the large sample size ensured sufficient statistical power.

Finally, the interval between assessment early in the acute care pathway and the occurrence of delirium during hospitalisation may have varied considerably, particularly among patients with longer hospital stays. An initially negative assessment may therefore have been appropriate at the time, with delirium developing later during admission. Future studies could incorporate length of hospital stay in sensitivity analyses to assess whether the performance of the different approaches varies according to duration of hospitalisation.

### Future perspectives

It would be beneficial to mandate the prehospital documentation of cognitive functioning in referral letters or ambulance reports of older patients conveyed to the emergency department, as it could potentially lead to earlier identification. While emergency departments have increasing focus on delirium recognition and there is a substantial body of literature on delirium in the emergency department, the prehospital/ambulance setting has been far less studied.

The Institute for Healthcare Improvement (IHI) recommends screening for delirium in acute care settings at least every 12 h [9]; however, earlier assessment, beginning at the first point of contact at home, or in ambulance, may be effective and relevant. Early recognition enables direct interventions, such as involving family members and ensuring the patient is in a suitable environment like a calm emergency department room. Delirium identification could be further enhanced through targeted training of emergency medical services personnel, integration of clinical decision support systems, or the application of natural language processing (NLP) to extract relevant information from clinical narratives [35]. Future studies comparing delirium identification approaches in the prehospital setting are needed to better understand how to improve early recognition and care in primary and emergency care pathways. The failure to identify delirium can lead to poor patient outcomes, resulting in harm from the very first hours of the patient journey (e.g. anxiety, dehydration, pressure sores) [36–38].

## Conclusion

As expected, all three approaches to delirium identification, the documented prehospital indicators, the VMS risk assessment, and 4AT screening, are associated with delirium during hospitalisation.

The documented prehospital indicators could be of added value, and we see this as a starting point for research into how this information could be used in the acute care pathway for older patients with or at risk for delirium.

## Supplementary Information

Below is the link to the electronic supplementary material.

Supplementary file1 (DOCX 655 KB)

## Data Availability

The datasets generated and analysed during the current study contain sensitive patient information and cannot be made publicly available due to ethical and legal restrictions. All data belong to the Acutelines biobank; researchers can contact Acutelines via acutelines@umcg.nl for more information. Access to anonymised data may be granted to qualified researchers upon reasonable request to the corresponding author with additional permission of the Acutelines biobank, subject to approval by the institutional ethics committee and execution of appropriate data sharing agreements.

## Abbreviations

4AT: 4 A’s test
AMT4: Abbreviated mental test-4
APOP: Acutely presenting older patient
CCI: Charlson comorbidity index
DOSS: Delirium observation screening scale
DSM-5: Diagnostic and statistical manual on mental disorders, Fifth Edition
ED: Emergency department
FAIR: Findable, accessible, interoperable, reusable
GDPR: General Data protection regulation
IQR: Interquartile range
OR: Odds ratio
REDCap: Research electronic data capture
SD: Standard deviation
SPSS: Statistical packages for social sciences
UMCG: University Medical Center Groningen
VMS: Veiligheidsmanagementsysteem (safety management system)
