# Supplementary material for: Comparing three approaches to delirium identification in older patients presenting to the emergency department
Source: Eur Geriatr Med. 2026 Oct 5;17(5):2705–13. doi: 10.1007/s41999-026-01612-w (PMC13755039; doi:10.1007/s41999-026-01612-w)
Supplement: Supplementary file 1 [file 41999_2026_1612_MOESM1_ESM.docx]

**Supplements**

**Supplement 1**

**Diagnostic and Statistical Manual of Mental Disorders (DSM-5) criteria for delirium**

A. A disturbance in attention (i.e., reduced ability to direct, focus, sustain, and shift attention) and awareness (reduced orientation to the environment).

B. The disturbance develops over a short period of time (usually hours to a few days), represents a change from baseline attention and awareness, and tends to fluctuate in severity during the course of a day.

C. An additional disturbance in cognition (e.g., memory deficit, disorientation, language, visuospatial ability, or perception).

D. The disturbances in Criteria A and C are not explained by another preexisting, established, or evolving neurocognitive disorder and do not occur in the context of a severely reduced level of arousal, such as coma.

E. There is evidence from the history, physical examination, or laboratory findings that the disturbance is a direct physiological consequence of another medical condition, substance intoxication or withdrawal (i.e., due to a drug of abuse or to a medication), or exposure to a toxin, or is due to multiple etiologies.

**Supplement 2**

**VMS, Safety Management System guidelines**

Positive in case of a total score of 1 or more on the following questions asked to the patient:

1. Do you have memory problems? (1 point)
2. Were you in need for self-care help in the last 24 hours? (1 point)
3. During previous admissions or illness, did you experience a transient state of confusion? (1 point)

**Supplement 3**

**4AT**

**Supplement 4**

**Standards for Reporting Diagnostic accuracy studies - STARD**

|  | **Section & Topic** | **No** | **Item** | **Reported on page #** |
| --- | --- | --- | --- | --- |
|  |  |  |  |  |
|  | **TITLE OR ABSTRACT** |  |  |  |
|  |  | **1** | Identification as a study of diagnostic accuracy using at least one measure of accuracy  (such as sensitivity, specificity, predictive values, or AUC) | 1 |
|  | **ABSTRACT** |  |  |  |
|  |  | **2** | Structured summary of study design, methods, results, and conclusions  (for specific guidance, see STARD for Abstracts) | 1 |
|  | **INTRODUCTION** |  |  |  |
|  |  | **3** | Scientific and clinical background, including the intended use and clinical role of the index test | 2 |
|  |  | **4** | Study objectives and hypotheses | 2 |
|  | **METHODS** |  |  |  |
|  | *Study design* | **5** | Whether data collection was planned before the index test and reference standard  were performed (prospective study) or after (retrospective study) | 2 |
|  | *Participants* | **6** | Eligibility criteria | 2 |
|  |  | **7** | On what basis potentially eligible participants were identified  (such as symptoms, results from previous tests, inclusion in registry) | 2 |
|  |  | **8** | Where and when potentially eligible participants were identified (setting, location and dates) | 2 |
|  |  | **9** | Whether participants formed a consecutive, random or convenience series | 2 |
|  | *Test methods* | **10a** | Index test, in sufficient detail to allow replication | 3 |
|  |  | **10b** | Reference standard, in sufficient detail to allow replication | 3 |
|  |  | **11** | Rationale for choosing the reference standard (if alternatives exist) | 3 |
|  |  | **12a** | Definition of and rationale for test positivity cut-offs or result categories  of the index test, distinguishing pre-specified from exploratory | 3 |
|  |  | **12b** | Definition of and rationale for test positivity cut-offs or result categories  of the reference standard, distinguishing pre-specified from exploratory | 3 |
|  |  | **13a** | Whether clinical information and reference standard results were available  to the performers/readers of the index test | 3 |
|  |  | **13b** | Whether clinical information and index test results were available  to the assessors of the reference standard | 3 |
|  | *Analysis* | **14** | Methods for estimating or comparing measures of diagnostic accuracy | 3 |
|  |  | **15** | How indeterminate index test or reference standard results were handled | 3 |
|  |  | **16** | How missing data on the index test and reference standard were handled | 3 |
|  |  | **17** | Any analyses of variability in diagnostic accuracy, distinguishing pre-specified from exploratory | 3 |
|  |  | **18** | Intended sample size and how it was determined | Suppl |
|  | **RESULTS** |  |  |  |
|  | *Participants* | **19** | Flow of participants, using a diagram | 4 |
|  |  | **20** | Baseline demographic and clinical characteristics of participants | 3, 4 |
|  |  | **21a** | Distribution of severity of disease in those with the target condition | - |
|  |  | **21b** | Distribution of alternative diagnoses in those without the target condition | - |
|  |  | **22** | Time interval and any clinical interventions between index test and reference standard | - |
|  | *Test results* | **23** | Cross tabulation of the index test results (or their distribution)  by the results of the reference standard | 4 |
|  |  | **24** | Estimates of diagnostic accuracy and their precision (such as 95% confidence intervals) | 4 |
|  |  | **25** | Any adverse events from performing the index test or the reference standard | - |
|  | **DISCUSSION** |  |  |  |
|  |  | **26** | Study limitations, including sources of potential bias, statistical uncertainty, and generalisability | 5,6 |
|  |  | **27** | Implications for practice, including the intended use and clinical role of the index test | 5,6 |
|  | **OTHER INFORMATION** |  |  |  |
|  |  | **28** | Registration number and name of registry | 6 |
|  |  | **29** | Where the full study protocol can be accessed | - |
|  |  | **30** | Sources of funding and other support; role of funders | 7 |
|  |  |  |  |  |

**Supplement 5**

Table: Baseline characteristics of patients with or without a completed 4AT

| ***Variables*** | ***No 4AT present N=2605*** | ***4AT present***  ***N=978*** | ***P**** |
| --- | --- | --- | --- |
| *Age, median (IQR)* | *76 (72-81)* | *76 (74-81)* | *<0.05* |
| *Female gender, n (%)* | *1139 (43.7%)* | *379 (38.8%)* | *<0.05* |
| *Marital status married n (%)* | *1019 (39.1%)* | *590 (60.3%)* | *<0.05* |
| *Living situation together* | *940 (36.1%)* | *568 (58.1%)* | *<0.05* |
| *Highest level of education*  Higher education, n (%) | *365 (14%)* | *224 (23%)* | *<0.05* |
| *Charlson Comorbidity Index (CCI)* | *5 (4-7)* | *5 (4-7)* | *0.77* |
| *APOP - Risk of functional decline (N=455)* | *(N=126)* | *(N=329)* |  |
| *Risk of functional decline percentage, median (IQR)* | *30 (20-40)* | *27 (18-37)* | *0.130* |
| *APOP - Indication for cognitive disorders* |  |  |  |
| *Indication for cognitive disorders* | *42/960* | *27/893* | *<0.05* |

**Supplement 6**

**A	B	C**

Figure 1. ROC-curves; A: documented prehospital indicators of delirium, B: VMS, C: 4AT

**Supplement 7**

**Table: Overlap in positive classifications between delirium identification approaches**

| Documented prehospital indicators of delirium | VMS positive | 4AT positive | n (%) |
| --- | --- | --- | --- |
| − | − | − | 169 (25.8) |
| + | − | − | 7 (1.4) |
| − | + | − | 179 (36.1) |
| − | − | + | 28 (5.6) |
| + | + | − | 24 (4.8) |
| + | − | + | 6 (1.2) |
| − | + | + | 55 (11.1) |
| + | + | + | 28 (5.6) |
| *Total 496, Missing 159. Total 655* | | | |

**Supplement 8**

**Examples of information from hospital charts**

Example 1: Reports seeing small animals and exhibits picking behaviour

Example 2: Doctor: delirium with hallucinations

Example 3: Geriatrician: delirium present
